# Dementia Knowledge Assessment Points to Need for Greater Understanding of Modifiable Risk Factors in Rural Areas

**DOI:** 10.1093/geroni/igaf122.2917

**Published:** 2025-12-31

**Authors:** M C Ehlman, Suzanne Leahy, Laurel Standiford Reyes, Reagan Lawrence

**Affiliations:** University of Nevada, Reno, Evansville, Nevada, United States; University of Southern Indiana, Evansville, Indiana, United States; University of Southern Indiana, Evansville, Indiana, United States; University of Southern Indiana, Evansville, Indiana, United States

## Abstract

Current lifetime risk of dementia is higher than previously thought (Fang, 2025), reinforcing the importance of public health initiatives focusing on brain health across the lifespan. The aim of this project was to assess baseline knowledge about Alzheimer’s disease (AD) in a mostly rural Midwest region to inform educational initiatives among a network of eight Dementia Friendly Communities (DFC). A public university supported the network development as a part of its Geriatric Workforce Enhancement Program funded by the United States Health Resources and Services Administration (HRSA), designed to impact rural communities. The regional assessment surveyed 159 community members and healthcare professionals from 11 regional counties, using Weise’s 30-item Basic Knowledge of Alzheimer’s Disease, a survey instrument validated for use with rural participants. The assessment was conducted in two stages. Local purposive samples were gathered by network DFCs later supplemented by convenience sampling at regional rural dementia workshops. The sample was 77.4% rural and medically underserved as defined by HRSA. Consistent with previous literature, 64.8% of participants responded that “Persons with a history of diabetes or high blood pressure are at greater risk of AD” (35.2% answered incorrectly). Additional gaps in knowledge were identified where respondents answered false (incorrectly) that “There may be a link between serious head injury and AD” (32.1% answered incorrectly) and “Healthy eating may decrease your AD risk” (26.4% answered incorrectly). Results highlight the need to examine the theoretical underpinnings linking brain health knowledge and positive health behaviors in rural areas to address modifiable risk factors.

